# Safety and Tolerability of Continuous Inhaled Iloprost Therapy for Severe Pulmonary Hypertension in Neonates and Infants

**DOI:** 10.3390/children11060703

**Published:** 2024-06-07

**Authors:** Amit V. Krishnan, Victoria Freniere, Rakesh Sahni, Diana P. Vargas Chaves, Sankaran S. Krishnan, Dimitrios Savva, Usha S. Krishnan

**Affiliations:** 1 Department of Pediatrics (Cardiology), Columbia University Irving Medical Center, New York, NY 10932, USA; akrishna@student.touro.edu; 2 Department of Pharmacy, New York-Presbyterian Hospital, New York, NY 10065, USA; victoria.freniere@bmc.org (V.F.); dis9082@nyp.org (D.S.); 3 Department of Pediatrics (Neonatology), Columbia University Irving Medical Center, New York, NY 10032, USA; rs62@cumc.columbia.edu (R.S.); dv2373@cumc.columbia.edu (D.P.V.C.); 4 Department of Pediatrics, New York Medical College, Valhalla, NY 10595, USA; sankaran_krishnan@nymc.edu

**Keywords:** continuous iloprost, pulmonary hypertension, neonate, infant, outcome

## Abstract

This is a single-center retrospective study to assess the safety and tolerability of continuous inhaled iloprost use as rescue therapy for refractory pulmonary hypertension (PH) in critically ill neonates and infants. A retrospective chart review was performed on 58 infants and data were collected at baseline, 1, 6, 12, 24, 48 and 72 h of iloprost initiation. Primary outcomes were change in heart rate (HR), fraction of inspired oxygen (FiO_2_), mean airway pressures (MAP), blood pressure (BP) and oxygenation index (OI). Secondary outcomes were need for extracorporeal membrane oxygenation (ECMO) and death. 51 patients treated for >6 h were analyzed in 2 age groups, neonate (≤28 days: n = 32) and infant (29–365 days: n = 19). FiO_2_ (*p* < 0.001) and OI (*p* = 0.01) decreased, while there were no significant changes in MAP, BP and HR. Of the fifteen patients placed on ECMO, seven were bridged off ECMO on iloprost and eight died. Twenty-four out of fifty-one patients (47%) recovered without requiring ECMO, while twelve (23%) died. Iloprost as add-on therapy for refractory PH in critically ill infants in the NICU has an acceptable tolerability and safety profile. Large prospective multicenter studies using iloprost in the neonatal ICU are necessary to validate these results.

## 1. Introduction

Pulmonary hypertension (PH) is a severe and often fatal disease, especially when it occurs in the neonatal and infant age group. PH can present as persistent pulmonary hypertension of the newborn (PPHN) in preterm or term neonates. PPHN can occur either as primary or idiopathic PPHN in about 10% neonates and secondary, in association with acute respiratory inflammation in the setting of meconium aspiration syndrome, pneumonias, hyaline membrane disease, transient tachypnea of newborn, developmental lung diseases, genetic disorders, as well as in congenital heart diseases (CHDs) [1,2,3]. Neonates with primary PPHN have an abnormal transition from fetal to postnatal circulation, resulting in sustained elevations of pulmonary vascular resistance and persistent hypoxemia after birth compromising hemodynamics [2,3]. Preterm infants with ongoing bronchopulmonary dysplasia (BPD) or term infants with CHD or other developmental lung disorders can present with significant and persistent PH even after the first few months of life and can have significant morbidity and mortality from PH crises in association with infection and inflammation [3,4].

Therapies for PH target the components of vascular remodeling and belong to three classes: phosphodiesterase inhibitors, endothelin receptor antagonists, and prostanoids [4,5,6]. Prostanoids are analogs of prostacyclin, which when bound to receptors, cause increases in cyclic adenosine monophosphate that result in a cascade of phosphorylation events that subsequently result in smooth muscle relaxation and reduced cell proliferation, thereby reducing pulmonary vascular resistance and inhibiting the remodeling associated with PH [6,7,8,9,10,11,12,13]. Although inhaled nitric oxide (iNO) is recommended as first line therapy to decrease PVR in infants with PPHN, and is the only approved PH therapy for PPHN, it does not improve survival and about 40% of neonates may fail to respond to iNO alone [7]. Epoprostenol, the first approved prostanoid medication, significantly improved long-term survival in PH patients. It has also been used successfully in neonatal PH [8,9]. Epoprostenol is administered as a continuous intravenous infusion through an indwelling central line, which is associated with a host of additional risks, such as need for access, bacteremia, sepsis, thromboembolism and rebound PH if the infusion is interrupted due to its extremely short half-life [6]. Iloprost is a more chemically stable prostaglandin analog and may be administered intermittently or continuously via inhalation. The inhaled administration specifically targets the lung vessels with less systemic effects and possibly less ventilation perfusion mismatch than intravenous epoprostenol, especially in infants with developmental lung disorders [6,7,8,9,10,11,12,13]. Iloprost peaks at 20–30 min and leaves the system within an hour after administration. Thus, continuous iloprost inhalation to prevent fluctuations in PA pressures is an attractive option.

Several adult studies and a few small pediatric studies have demonstrated favorable outcomes when patients were placed on adjunctive inhaled iloprost therapy after they had inadequate responses to current PAH therapies. Although these studies suggested some benefits, they were smaller in size, did not have a uniform population base and did not look at the effects of continuous inhaled iloprost therapy in neonates and infants [12,13,14]. Neonates with PH may be refractory to iNO and may rapidly deteriorate, leading to extracorporeal membrane oxygenation (ECMO) cannulation and/or death. Intravenous or inhaled prostanoids have been used to try and maintain hemodynamics by lowering the PVR in these patients and act as additional therapy to iNO [5,6,7,8,9,10,11,12,13,15,16]. Evidence demonstrates that inhaled iloprost therapy significantly reduces pulmonary arterial pressure and PVR with equal potency to iNO with minimal systemic side effects within the pediatric population with CHD [11,14]. Little is known about the safety and efficacy of inhaled iloprost in the neonatal and infant population. The use of continuous iloprost in older children has recently been described [10]. The aim of this study is to evaluate the safety, tolerability and outcomes, including ECMO and death after continuous inhaled iloprost in infants with refractory PH in the NICU. We also describe the institutional practices for medication dosing, delivery, titration and wean of inhaled continuous iloprost therapy.

## 2. Materials and Methods

This is a single-center retrospective observational study of neonates and infants admitted to the NICU at Morgan Stanley Children’s Hospital of New York Presbyterian. Patients were included if they received iloprost therapy between February 2020 and May 2023. Patients < 1 year of age with the 6th World Symposium of Pulmonary Hypertension (6th WSPH) Groups I and III PH were included, while patients with Group II PH (left heart disease and pulmonary vein stenosis) and Group V PH with complex congenital heart disease were excluded. This study was approved by the Columbia University Irving Medical Center’s Institutional Review Board and electronic medical and pharmacy records were queried to identify patients and collect data. Since our new electronic Medical Record system, Epic, was implemented in February 2020, we could only consistently obtain data from this date and beyond.

Demographic data included age and weight at iloprost start, gestational age, birth weight, Apgar scores, iloprost dose and duration, iNO dose, inotropes, other PH medications, steroid administration, level of ventilatory support, need for ECMO, underlying diagnosis associated with PH, and outcomes. Additionally, fraction of inspired oxygen (FiO_2_), mean airway pressure (MAP), partial pressure of arterial oxygen (PaO_2_), mean blood pressure (mBP), heart rate (HR) and oxygen index ((OI) = FiO_2_ × MAP/PaO_2_) were collected at baseline, 1, 6, 12, 24, 48 and 72 h after initiation of iloprost therapy. The vasoactive inotrope score (VIS) was calculated using the doses of inotropes at the time of iloprost start (Dopamine dose + Dobutamine dose + 100 × Epinephrine dose + 10 × Milrinone dose + 100 × Norepinephrine dose (all in mcg/kg/minute) + 10,000 × Vasopressin dose (U/kg/min)) [17,18].

### 2.1. Method of Administration

Inhaled iloprost was only used in infants with severe persistent PH despite treatment with iNO and other PH therapy. A test dose of iloprost (2.5 mcg) was administered over 20 min to assess for hypotension or worsening in oxygenation. If the initial iloprost dose was well tolerated, continuous therapy was initiated. Iloprost was administered through the inhalation tubing port closest to the endotracheal tube (to reduce dead space) and occasionally extra diluent was provided to prevent crystallization or clogging of the tube. The treatment dose range of iloprost was between 1 and 7.5 mcg/h. Our weaning protocol consisted of a gradual reduction in the dosage to 1 mcg/h. This was followed by weaning to intermittent iloprost with bolus dosing at gradually prolonged intervals up to every four hours until discontinuation. Primary outcomes included change in FiO_2_, MAP, OI, HR and mBP from baseline to 72 h. Secondary outcomes included the need for ECMO or death during iloprost therapy. The reason for stopping iloprost and side effects were documented.

The data were analyzed as a whole, and patients were separated into two groups—neonates (<28 days) and infants (28–365 days)—as the etiology of PH and the frailty of the patients are different in the two groups.

### 2.2. Statistical Analysis

The data were analyzed using SPSS version 16 for Windows. Binomial and categorical data were analyzed by chi-squared and Fisher’s exact test. Non-parametrically distributed continuous data were analyzed by the Mann–Whitney U test. Continuous variables were not assumed to be normally distributed, so values were reported as medians with interquartile range (IQR) provided. A *p* value of <0.05 was considered significant. Friedman’s two-way ANOVA by ranks was used to evaluate distributions of measured pulmonary and systemic respiratory and hemodynamic markers (FiO_2_, MAP, OI, mean BP, HR) over time, with a *p* value of <0.05 considered significant. 

## 3. Results

### 3.1. Demographics 

A cohort of fifty-one patients with the sixth WSPH Group I or III PH who were treated with inhaled iloprost as rescue therapy were analyzed. Seven additional patients received iloprost for <6 h and were not included in the analysis. The patients ranged from 0 to 310 days old, and their weights ranged from 2.9 to 4.3 kg. Within this cohort, 31 patients survived, while 20 died. Fifteen patients required ECMO during their hospital course. Thirty-two patients ≤28 days of age at the time of treatment were categorized into the neonate group and the remaining nineteen patients ≥28–365 days of age at time of treatment were categorized into the infant group. Thirty-one neonates were ≤10 days of age, while one patient was 21 days old with a diagnosis of surfactant protein deficiency. In the neonate group, PH was secondary to congenital heart disease (CHD) in 10 patients, and congenital diaphragmatic hernia (CDH) in nine. Other causes of PH in this group included meconium aspiration, hypoxic ischemic encephalopathy, sepsis and developmental lung diseases. The infants were 41–310 days old at time of iloprost therapy and had a diagnosis of CHD in 12, CDH in three, and BPD in four. Gender differences in demographics and clinical characteristics of the study population are described in Table 1. There were no statistically significant differences in demographics, clinical characteristics, and outcomes between male and female neonates.

There was a significantly greater proportion of neonates in our sample compared to infants (Table 2). There was also a difference in gestational age (the BPD patients in the infant group had a lower gestational age and presented with severe PH later), weight at time of iloprost dosing, and in VIS between the two groups. Two infants with critical PH had a VIS of 0. This was secondary to vasoactive medications being discontinued after being cannulated on ECMO.

### 3.2. Other PH Medications

All patients were on 20 PPM iNO, 12 patients received sildenafil, four received sildenafil and bosentan (dual therapy), nine in the neonate group were on prostaglandin E1 (to keep the ductus arteriosus patent) and three were on parenteral epoprostenol, which was weaned off when iloprost was initiated. Inotropes at the time of iloprost start included milrinone (32), epinephrine (30), vasopressin (19), dopamine (9), dobutamine (6) and norepinephrine (5), demonstrating the severity of hemodynamic compromise among these children.

There was a significantly greater proportion of patients in our sample who were not cannulated to ECMO compared to patients who did (Table 3). There was a significant difference in Apgar scores at 5 min between ECMO and non-ECMO patients, but none of the other parameters achieved significance.

There was a significant difference in the proportion of patients who survived compared to the proportion of patients who died while on iloprost in our sample (Table 4). No other differences were observed between surviving and deceased patients.

### 3.3. Improvement in Parameters over the Evaluated Time Period

**Figure 1:**
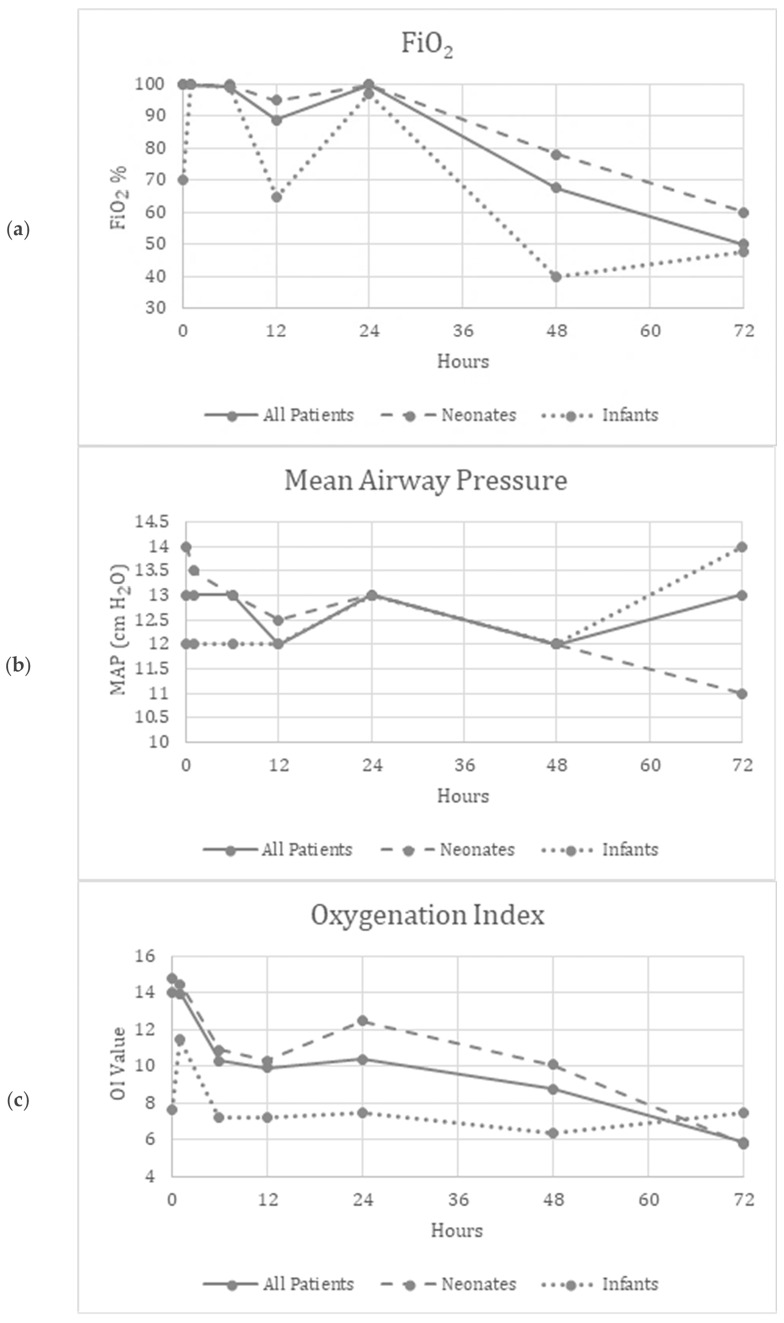

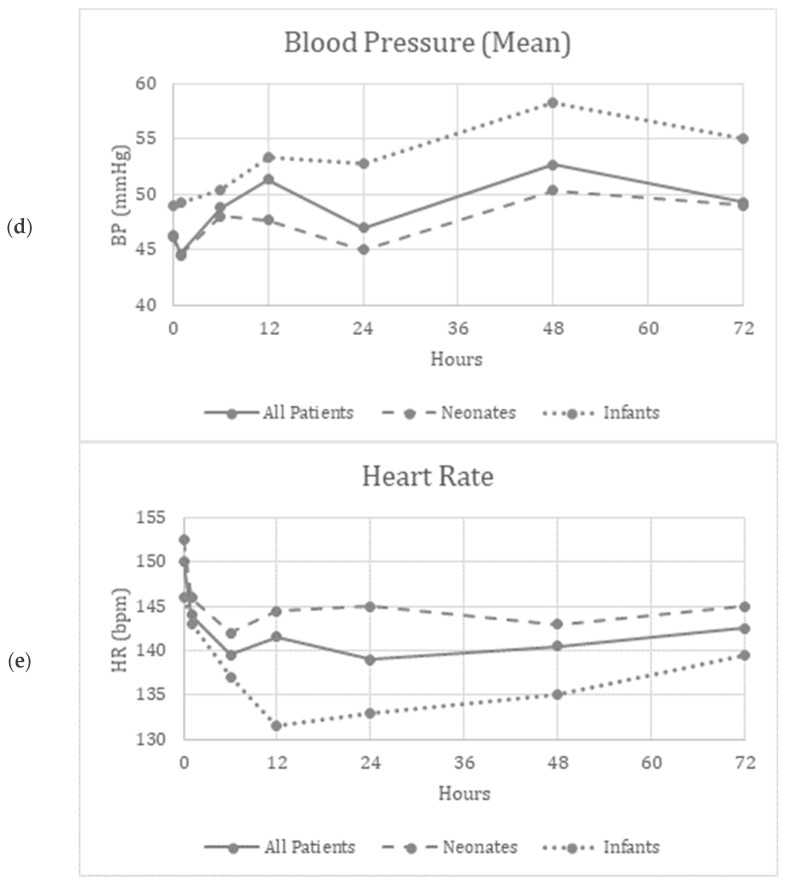
(**a**–**e**) Graphical depiction of the physiologic parameters measured over time in all patients, neonates and infants.

Friedman’s two-way analysis of variance was performed to detect significant changes in FiO_2_, OI, MAP, mean BP and HR over the 72 h study period. FiO_2_ (*p* < 0.001) and OI (*p* = 0.01) decreased over the study period, suggesting improved oxygenation (Figure 1a,c). Supplementary Figure S1a,c show similar improvements in the patients who did not need ECMO.

### 3.4. Safety and Tolerability Data

There were no significant differences reported in the MAP (*p* = 0.34), mBP (*p* = 0.12), or HR (*p* = 0.97) over the study period, suggesting the safety and tolerability of continuous inhaled iloprost in these patients (Figure 1b,d,e). Supplementary Figure S1b,d,e show similar trends in patients who did not need ECMO.

### 3.5. Patients Who Received Iloprost <6 h (Excluded from Data Analysis)

Seven patients received iloprost for <6 h; four out of seven patients had acute deterioration prompting ECMO cannulation and iloprost discontinuation within the 6 h time period. Two patients did not have evident improvement in oxygenation or hemodynamics with the test dose and one patient had hypotension with severe sepsis.

### 3.6. Side Effects

No significant side effects were charted. Note that the usual side effects of headaches seen in older patients could not be documented. There was no significant bronchospasm documented with iloprost administration in any patient or need for bronchodilators during the study period. Due to the gradual weaning protocol and background iNO therapy, no rebound PH was noted in our patients. There were no instances of pulmonary hemorrhage or other bleeding attributed to iloprost in this cohort.

## 4. Discussion

Inhaled prostanoids like iloprost have several advantages over intravenous prostanoids, including the avoidance of significant ventilation perfusion mismatch, potential attenuation of systemic side effects and bypassing vascular access availability or medication incompatibility issues [11,12,19,20,21]. Subcutaneous prostanoids have been used in this age group; however, pain issues and pump shortage have recently significantly impacted this mode of delivery in the neonatal unit. Inhaled iloprost has the advantage over inhaled epoprostenol because of its longer half-life and stability [6,8]. This medication is often used as rescue therapy in infants with refractory PH despite maximal iNO. Inhaled iloprost is delivered using a syringe pump attached to the port closest to the endotracheal or tracheostomy tube through the inspiratory portion of the ventilator circuit. This is performed to reduce dead space and prevent crystallization in the tubing. In the current study, we have described the methodology of use of continuous iloprost and studied the safety, tolerability and short-term efficacy of the medication in critically ill neonates and infants in a single-institution NICU with refractory WSPH Group 1 or Group 3 PH. 

This is the first case series describing the use of continuous iloprost in a NICU cohort with serial evaluation over a 72 h time period. A recent publication by Colglazier et al. describes the methodology of continuous iloprost, which is very similar to our unit but differs from our study in that they were older patients, and the study described the very early response over 30 min of iloprost delivery. Our study is limited the infant age group (<1 year of age) and those with iloprost therapy > 6 h, thus describing the short-to-medium term use of the medication in patients under 1 year of age. Our side effect profile is similar to their study in that continuous iloprost was not associated with significant bronchospasm or any other significant side effects of prostanoids. All our patients on whom iloprost was started were considered refractory PH, already on 20 PPM of iNO (our maximum dose) and hence, we did not compare the two inhaled medications.

Additionally, patient demographics, clinical characteristics and outcomes were reported and stratified with respect to sex (M/F), age (neonate/infant), clinical characteristics (ECMO/No ECMO) and outcomes (survived/deceased) (Table 1, Table 2, Table 3 and Table 4). There are several neonatal studies which have suggested male sex as a risk factor for higher incidence of BPD, developmental lung diseases as well as response to therapy; however, in our limited subset, a significant difference was not noted between male and female patients [22,23,24]. There were very few statistically significant differences between these stratified groups, suggesting that their impact on the efficacy of iloprost is minimal. Verma et al. recently described the potential for inhaled iloprost as a treatment option for PPHN. The trial included 22 neonates with PPHN on intermittent iloprost who did not respond to iNO alone, of whom 55% were considered responders and 45% non-responders who were placed on ECMO or died [12]. This study was limited to PPHN in the neonatal period, which, when not associated with other disorders, usually resolves in 2–3 weeks. Our study includes a whole range of patients with refractory PH secondary to multiple etiologies in the neonatal and infant age groups.

It is possible that the continuous mode of administration reduces the swings in pulmonary and systemic hemodynamics following medication dosage, providing sustained pulmonary vasodilatory effect and minimizing acute blood pressure variations in this fragile population. Additionally, iloprost has also been successfully used as part of strategies to wean and bridge off ECMO support by specifically targeting PH and therefore reducing right ventricular afterload and improving cardiac output. Other studies have speculated that the addition of iloprost to iNO utilizes both the cyclic adenosine monophosphate and cyclic guanosine monophosphate pathways and provides a synergistic effect [10,25]. Since all of our patients were already on maximal doses of iNO prior to initiation of iloprost therapy, the contribution of a synergistic effect in the improved oxygenation status among our patients cannot be established. It is possible that higher doses of iloprost (5–7.5 mcg/h) may have more sustained improvements in PH but our patient numbers and duration of therapy on higher doses were too small to analyze potential effects. Other available inhaled prostanoids include inhaled treprostinil, which is not possible in ventilated patients, but is an attractive option in the outpatient setting. Additionally, some centers use inhaled epoprostenol, using the same preparation as used in the intravenous route, but there is speculation that this preparation may not be suitable for inhaled administration due to potential inflammatory response in the airways and lung parenchyma induced by components in the formulation of this medication [19,20,21].

As for all infant and pediatric studies, this study is also limited by small numbers, its retrospective format and lacks placebo controls. These were not possible given the patient population being critically ill and iloprost being administered as a rescue medication for refractory PH. Although rSO2 (NIRS) data were also collected, there were multiple missing values precluding meaningful analysis in the current study. Most infants who were cannulated on veno-arterial ECMO had their inotropes stopped, which would impact the significance of VIS scores, especially for patients who were started on iloprost while on ECMO. Thus, the VIS score was not useful in this study. Additionally, in some infants (especially the seven who received iloprost <6 h), iloprost was started during rapid hemodynamic deterioration while being evaluated for ECMO cannulation and the medication was stopped after cannulation on ECMO by physician preference, limiting longer-term evaluation over time in this group. Iloprost was started in a few patients to reduce PH while bridging off ECMO, but the numbers were not adequate for meaningful analysis. A future protocolized strategy of iloprost use in these patients will provide valuable data.

## 5. Conclusions

This is the largest study describing the use of continuous inhaled iloprost in critically ill neonates and infants with severe pulmonary hypertension. Inhaled iloprost is safe, well tolerated and appears to improve pulmonary and systemic hemodynamics in critically ill infants with PH who usually face very high mortality. Additionally, inhaled iloprost provides an attractive alternate pathway to administer prostanoids. It may also help stabilize some patients prior to ECMO and has the potential to assist in bridging off ECMO in selected patients. Larger prospective multicenter studies are necessary to study the use of this medication in improving outcomes in the infant and pediatric age groups.

## Figures and Tables

**Table 1:** Demographic and clinical characteristics of the study population stratified by sex.

	Number of Patients (%)	Male (%)	Female (%)	*p* *
Total	51 (100%)	30	21	
Neonate	32 (62.7%)	17 (56.7%)	15 (71.4%)	0.28
Need for ECMO	15 (29.4%)	8 (26.7%)	7 (33.3%)	0.61
Mortality	20 (39.2%)	14 (46.7%)	6 (28.6%)	0.19
	Total (range)	Male (IQR)	Female (IQR)	*p* **
Median Birth Weight (kg)	3.080 (0.375–4.420)	3.13 (2.320–3.380)	3.08 (2.550–3.300)	0.69
Median Gest. Age (Weeks)	38.57 (23–41)	37.57 (35.71–39.00)	38.71 (37.14–39.14)	0.28
Median Weight at Iloprost Admin. (kg)	3.37 (2.06–7.3)	3.43 (2.99–4.38)	3.30 (2.97–3.71)	0.70
Apgar Score 1¢	6.00 (2.00–8.00)	5.00 (2.50–7.00)	7.00 (3.00–8.00)	0.25
Apgar Score 5¢	8.00 (7.00–9.00)	7.00 (7.00–8.50)	8.00 (7.00–9.00)	0.50
VIS	13.00 (0–19.00)	13.00 (3.50–16.80)	13.00 (6.00–20.00)	0.62
Iloprost # Days	3.00 (0.25–37)	3.00 (1.00–8.80)	3.00 (1.00–7.00)	0.76

* chi-squared test; ** Mann–Whitney test.

**Table 2:** Outcomes in neonates and infants with PH treated with iloprost (n = 51).

	Neonate (%)	Infant (%)	*p* *
Total	32 (62.7%)	19 (37.3%)	**0.01**
Male	17 (53.1%)	13 (68.4%)	0.28
ECMO	12 (37.5%)	3 (15.8%)	0.12 **
Deceased	13 (40.6%)	7 (36.8%)	0.79
	median (IQR)	Median (IQR)	*p* ***
Birth Wt (kg) (range)	3.195 (0.540–4.29)	3.05 (0.375–4.42)	0.52
Gest. age (weeks)	39.00 (37.14–39.14)	37.00 (30.14–38.79)	**0.02**
Weight at iloprost administration (kg)	3.21 (2.75–3.38)	4.53 (3.77–5.56)	**<0.01**
Apgar score 1¢	5.00 (2.75–8.00)	6.50 (2.75–8.00)	0.74
Apgar score 5¢	7.00 (6.50–9.00)	8.00 (7.00–9.00)	0.55
VIS	14.50 (6.75–23.13)	5.00 (0.00–14.50) *	**0.03**
Iloprost # days	1.00 (1.00–5.50)	3.00 (1.00–10.00)	0.07

* chi-squared test; ** Fischer’s exact test; *** Mann–Whitney test. Bold indicates significance.

**Table 3:** Characteristics of patients requiring vs. not requiring ECMO cannulation.

	ECMO (%)	No ECMO (%)	*p* *
Total	15 (29.4%)	36 (70.6%)	**<0.01**
Male	8 (53.3%)	22 (61.1%)	0.61
Neonate	12 (80.0%)	20 (55.6%)	0.12 **
Deceased	7 (46.7%)	13 (36.1%)	0.48
	ECMO (IQR)	No ECMO (IQR)	*p* ***
Birth Wt (kg)	3.08 (2.49–3.28)	3.13 (2.50–3.39)	0.88
Gest. Age (weeks)	38.57 (37.14–39.00)	38.21 (35.67–39.00)	0.88
Dosing Wt (kg)	3.22 (2.72–3.71)	3.51 (3.20–4.13)	0.10
Apgar score 1¢	6.00 (4.50–8.00)	5.00 (2.00–8.00)	0.23
Apgar score 5¢	9.00 (7.00–9.00)	7.00 (5.00–8.00)	**0.04**
VIS	15.00 (4.50–22.50)	10.50 (3.75–15.63)	0.22
Iloprost # days	2.00 (1.00–14.50)	3.00 (1.00–7.25)	0.55

* chi-squared test; ** Fisher’s exact test; *** Mann–Whitney test. Bold indicates significance.

**Table 4:** Characteristics of patients who survived vs. deceased patients.

	Survived (%)	Deceased (%)	*p* *
Total	31 (60.8%)	20 (39.2%)	**0.03**
Male	16 (51.6%)	14 (70.0%)	0.19
Neonate [<4 wk age]	19 (61.3%)	13 (65.0%)	0.79
ECMO	8 (25.8%)	7 (35.0%)	0.48
	Survived (IQR)	Deceased (IQR)	*p* **
Birth Wt (kg)	3.20 (2.50–3.58)	3.06 (2.41–3.28)	0.32
Gest. age (weeks)	39.00 (37.00–39.14)	37.29 (35.71–38.89)	0.23
Dose Wt (kg)	3.70 (3.21–4.41)	3.22 (2.75–3.61)	0.06
Apgar score 1¢	6.00 (3.00–8.00)	5.00 (2.50–7.50)	0.83
Apgar score 5¢	8.00 (7.00–9.00)	7.00 (7.00–9.00)	0.59
VIS	11.00 (3.00–16.50)	14.00 (6.00–21.25)	0.40
Iloprost # days	3.00 (1.00–10.00)	1.50 (0.94–6.75)	0.48

* chi-squared test; ** Mann–Whitney test. Bold indicates significance.

## Data Availability

Data are contained within the article and Supplementary Materials.

## Supplementary Materials

The following supporting information can be downloaded at: https://www.mdpi.com/article/10.3390/children11060703/s1, Figure S1a–e depict changes in FiO_2_, mean airway pressure, oxygenation index, mean blood pressure and heart rate in patients who did not need ECMO (continuous lines vs patients who needed ECMO (dotted line). The trends on patients who did not need ECMO are similar to all patients depicted in Figure 1. These values become not relevant once patients are placed on ECMO.
