# Supplementary material for: Safety and Tolerability of Continuous Inhaled Iloprost Therapy for Severe Pulmonary Hypertension in Neonates and Infants
Source: Children (Basel). 2024 Jun 7;11(6):703. doi: 10.3390/children11060703 (PMC11201391; doi:10.3390/children11060703)
Supplement: Supplementary file 1 [file children-11-00703-s001.zip › children-2998713-supplementary.pdf]

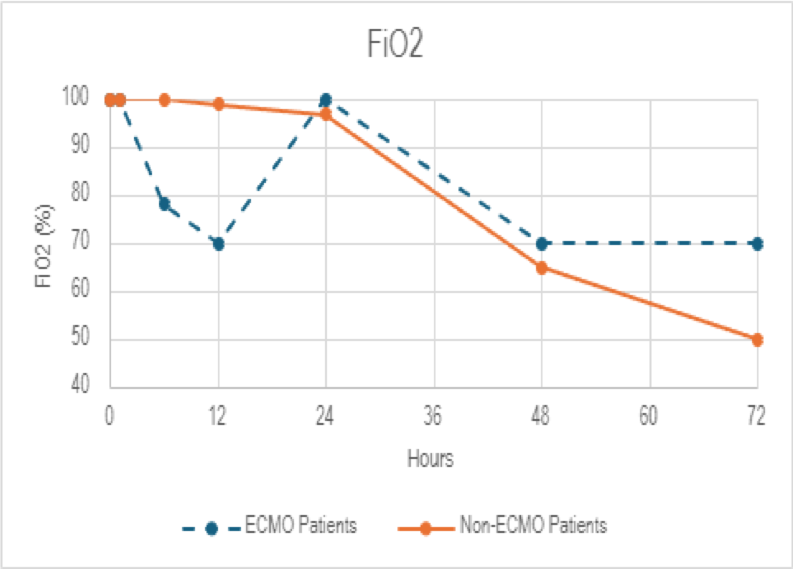

Figure S1a

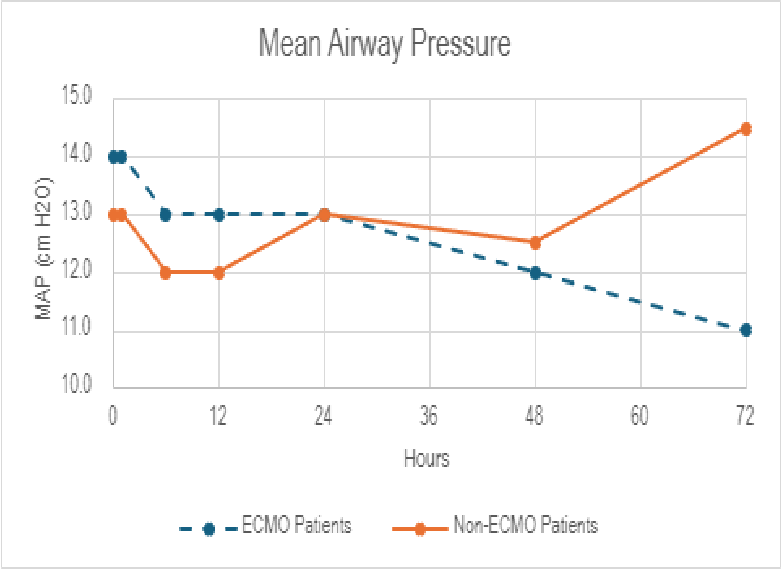

Figure S1b

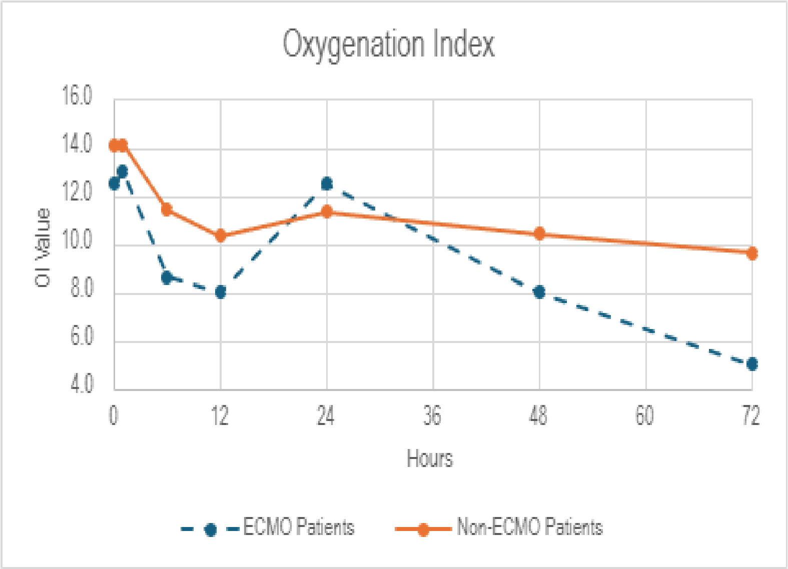

Figure S1c

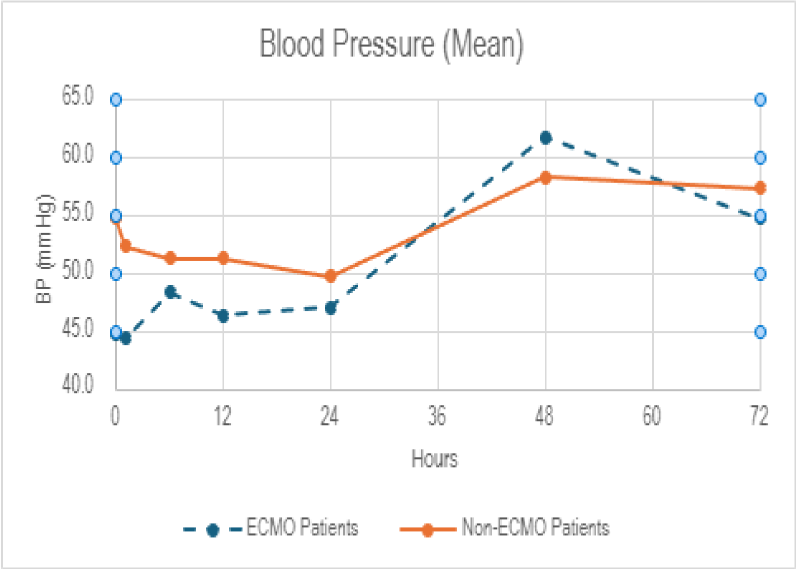

Figure S1d

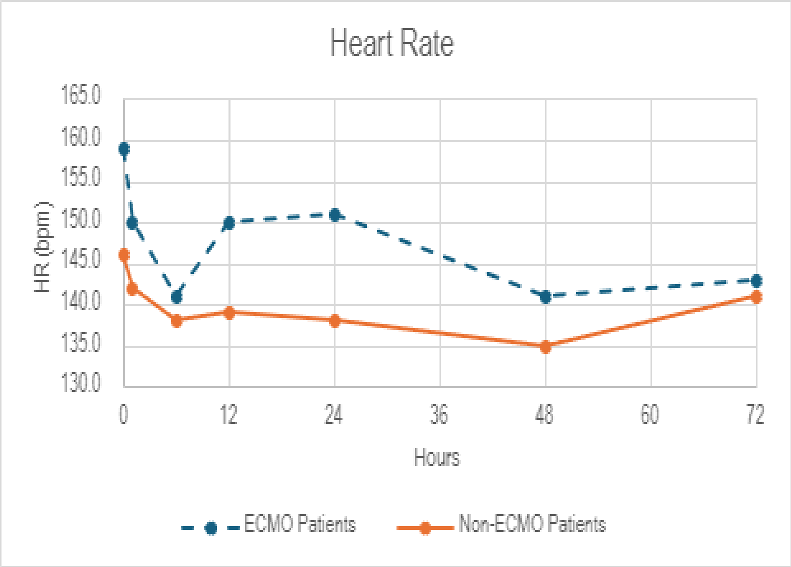

Figure S1e

Figure S1a-e depict changes in FiO2, mean airway pressure, oxygenation index, mean blood pressure and heart rate in patients who did not need ecmo (continuous lines vs patients who needed ecmo (dotted line). The trends on patients who did not need ecmo are similar to all patients depicted in Figure 1. These values become not relevant once patients are placed on ecmo.
